# Differential impact of dual-active ingredient long-lasting insecticidal nets on primary malaria vectors: a secondary analysis of a 3-year, single-blind, cluster-randomised controlled trial in rural Tanzania

**DOI:** 10.1016/S2542-5196(23)00048-7

**Published:** 2023-05-08

**Authors:** Nancy S Matowo, Manisha A Kulkarni, Louisa A Messenger, Mohamed Jumanne, Jackline Martin, Elizabeth Mallya, Eliud Lukole, Jacklin F Mosha, Oliva Moshi, Boniface Shirima, Robert Kaaya, Mark Rowland, Alphaxard Manjurano, Franklin W Mosha, Natacha Protopopoff

**Affiliations:** a Department of Disease Control, London School of Hygiene & Tropical Medicine, London, UK; b School of Epidemiology and Public Health, University of Ottawa, OT, Canada; c Department of Environmental and Occupational Health, School of Public Health, University of Nevada, Las Vegas, NV, USA; d Department of Parasitology, National Institute for Medical Research, Mwanza Medical Research Centre, Mwanza, Tanzania; e Kilimanjaro Christian Medical University College, Moshi, Tanzania

## Abstract

**Background:**

Gains in malaria control are threatened by widespread pyrethroid resistance in malaria vectors across sub-Saharan Africa. New long-lasting insecticidal nets (LLINs) containing two active ingredients (dual active-ingredient LLINs) have been developed to interrupt transmission in areas of pyrethroid resistance. We aimed to evaluate the effectiveness of three dual active-ingredient LLINs compared with standard pyrethroid LLINs against pyrethroid-resistant malaria vectors in rural Tanzania.

**Methods:**

In this study, we did a secondary analysis of entomological data from a four-group, 3 year, single-blind, cluster-randomised controlled trial carried out between Feb 18, 2019, and Dec 6, 2021. We conducted quarterly indoor mosquito collections using the Centers for Disease Control and Prevention light trap, in eight houses in each of the 84 study clusters in the Misungwi district, northwestern Tanzania. *Anopheles* vectors were then tested for malaria parasites and identified at species level, to distinguish between sibling species of the *Anopheles gambiae* and *Anopheles funestus* groups, using molecular laboratory techniques. The primary outcomes were density of different malaria vector species measured as the number of female *Anopheles* collected per household per night, the entomological inoculation rate (EIR), an indicator of malaria transmission, and sporozoite rate. Entomological outcomes were assessed on the basis of intention to treat, and the effect of the three dual active-ingredient LLINs was compared with the standard pyrethroid LLINs at household level.

**Findings:**

Dual active-ingredient LLINs had the greatest effect on *Anopheles funestus* sl, the most efficient vector in the study area, with comparatively weak effect on *An arabiensis*. *An funestus* density was 3∙1 per house per night in the pyrethroid LLIN group, 1∙2 in the chlorfenapyr pyrethroid LLIN group (adjusted density ratio [aDR]=0∙26, 95% CI 0∙17–0∙14, p<0∙0001), 1∙4 in the piperonyl-butoxide pyrethroid LLIN group (aDR=0∙49, 0∙32–0∙76, p=0∙0012), and 3∙0 in the pyriproxyfen pyrethroid LLIN group (aDR=0∙72, 0∙47–1∙11, p=0∙15). Malaria transmission intensity was also significantly lower in the chlorfenapyr pyrethroid group, with 0∙01 versus 0∙06 infective bites per household per night in the pyrethroid LLIN group (aDR=0∙21, 0∙14–0∙33, p<0∙0001). Ecological niche models indicated that vector-species distribution was stable following LLIN intervention despite the reductions observed in *An funestus* sl density.

**Interpretation:**

Chlorfenapyr pyrethroid LLINs were the most effective intervention against the main malaria vector *An funestus* sl over 3 years of community use, whereas the effect of piperonyl-butoxide pyrethroid LLIN was sustained for 2 years. The other vector, *An arabiensis*, was not controlled by any of the dual active-ingredient LLINs. Additional vector control tools and strategies targeted to locally prevalent vector species evading dual active-ingredient LLINs should be deployed to further reduce malaria transmission and achieve elimination.

**Funding:**

The Department for International Development, UK Medical Research Council, Wellcome Trust, the Department of Health and Social Care, and The Bill & Melinda Gates Foundation via the Innovative Vector Control Consortium.

## Introduction

The primary malaria-vector control tools, consisting of pyrethroid long-lasting insecticidal nets (LLINs) and indoor residual spraying, have substantially reduced malaria morbidity and mortality across sub-Saharan Africa.^1^ Among the challenges faced by malaria control strategies, the most important are widespread pyrethroid resistance in malaria-vector populations^1^ and insufficient funding for malaria control, which have led to intervention prioritisation by national malaria control programmes.

To address the biological challenge of insecticide resistance while sustaining malaria control gains, novel next-generation dual active-ingredient LLINs are under evaluation; some have received provisional recommendation by WHO.^1^, ^2^ Dual active-ingredient LLINs have shown better efficacy than standard pyrethroid-only LLINs on malaria indicators in several community randomised trials.^3^, ^4^, ^5^, ^6^ However, the effectiveness of vector control interventions may vary according to mosquito species with differing behaviours, ecologies, and epidemiological settings. *Anopheles* vector species composition might also change following intervention deployment, a phenomenon that has been reported previously.^7^

**Research in context**

**Evidence before this study**

Insecticide resistance in malaria vectors is widespread in many endemic settings, thus presenting a serious threat to the efficacy of vector-control tools, mainly long-lasting insecticidal nets (LLINs) and indoor residual spraying. To sustain the progress made against malaria and to improve vector-control tools, WHO recommends the development of new insecticidal products with different modes of action against resistant vectors. Piperonyl butoxide LLINs received interim WHO recommendation as a novel malaria vector-control tool after showing better protection than standard pyrethroid LLINs against malaria infection and transmission in two cluster randomised controlled trials (cRCTs) in Tanzania and Uganda. The current study is a follow-up of the recently published large-scale cRCT assessing the effectiveness and cost-effectiveness against malaria of three types of dual active-ingredient LLINs, compared with pyrethroid-only LLINs in Tanzania. We searched for published papers in English in PubMed on Feb 10, 2022, without restrictions on the date, using the terms “mosquito” or “vectors” and “*Anopheles gambiae* complex”, “*Anopheles arabiensis”*, or “the *Anopheles funestus* group”, and “malaria”, “entomological inoculation rate”, “randomised controlled trial”, “village trials”, and “long lasting insecticidal net” or net in combination with “chlorfenapyr”, piperonyl butoxide”, or “pyriproxyfen”. We found five cRCTs, one in Burkina Faso, one in Uganda, two in Tanzania, and one village trial in Mali, assessing the effectiveness of dual active ingredient LLINs with some entomological outcomes. In Burkina Faso, the entomological inoculation rate (EIR) was 51% lower in the pyriproxyfen pyrethroid LLIN group compared with the standard LLIN group (standard pyrethroid LLIN). In Uganda, at 25 months follow-up, reduction in density of malaria vectors was 62% in the piperonyl-butoxide pyrethroid LLINs compared with standard pyrethroid LLIN. The Tanzanian study showed that the indoor overall vector density and EIR were significantly reduced in the chlorfenapyr pyrethroid LLIN group by 57% and 85%, respectively, after 2 years of community use, and by 58% and 73%, respectively, in the piperonyl-butoxide PY group only in year 1, compared with the standard pyrethroid LLIN group. In the Tanzanian cRCT, the pyriproxyfen pyrethroid LLIN did not have a significant impact on entomological outcomes when compared with the pyrethroid LLIN. In Mali, the piperonyl-butoxide pyrethroid LLINs did not show any improved control on indoor *Anopheles arabiensis* population density when compared with standard permethrin LLINs; however, sporozoite rates were lower in the piperonyl-butoxide pyrethroid LLIN group than in the the permethrin LLIN group only during the rainy and high-malaria-transmission seasons. Here, we report the effect of dual active-ingredient LLINs on each of the main vector species and associated malaria transmission, over 3 years of net use.

**Added value of this study**

This is to our knowledge one of the few large-scale community trials looking at the effectiveness of next-generation dual active-ingredient LLINs on the density of different vector species. Malaria in sub-Saharan Africa is highly heterogeneous due to ecological and climatic variations. Currently, the three main vectors are *Anopheles coluzzii*, *An gambiae* ss, and *An funestus*, with *An funestus* becoming the most important in eastern and southern Africa. A fourth vector, *An arabiensis*, a secondary vector, is involved in residual malaria transmission because of its outdoor biting behaviour and considerable behavioural plasticity, allowing it to feed on both animal and human hosts. Anopheles mosquito species diversity and malaria-transmission dynamics have implications during the deployment of effective malaria vector-control strategies. In this study, we observed differential effects of dual active-ingredient LLINs on different malaria-vector species. Two dual active-ingredient LLINs, chlorfenapyr pyrethroid LLINs and piperonyl-butoxide pyrethroid LLINs, were most effective compared with standard pyrethroid LLINs at controlling *An funestus* population density in the southern part of the study area with no impact on *An arabiensis*, suggesting that targeted control could be optimised to specific species focusing on high-burden settings to ensure sustained malaria control. This study's entomological findings provide additional insights to understand the epidemiological results observed in the trial, but might also explain some differences between trials done in other sites where vector-species composition differed. Chlorfenapyr pyrethroid LLINs were the most effective dual active-ingredient LLINs, and the effect was sustained for 3 years.

**Implications of all the available evidence**

Previous randomised controlled field trials supported by small-scale experimental hut trials have demonstrated the improved effectiveness of dual active-ingredient LLINs compared with standard pyrethroid LLINs on entomological indices. In hut trials, chlorfenapyr pyrethroid LLINs were found effective against several vector species, including *An funestus*, *An gambiae*, and *Culex quinquefasciatus*. A Cochrane review reported a significant impact of piperonyl-butoxide pyrethroid LLINs mainly on *An gambiae* ss. One large community trial showed improved efficacy of pyriproxyfen pyrethroid LLINs against *An gambiae* ss. The current cRCT contributes to previous evidence, as the first community trial assessing differential impacts of dual active-ingredient LLINs on the major malaria-vector species in sub-Saharan Africa. It reveals a superior impact of dual active-ingredient LLINs containing chlorfenapyr and piperonyl butoxide, compared with standard pyrethroid LLINs, in reducing population density of the major malaria vector *An funestus*, thereby preventing human infectious bites. The protective efficacy of chlorfenapyr pyrethroid LLINs were sustained for 3 years, whereas piperonyl-butoxide pyrethroid LLINs lasted for 12 months. These dual active-ingredient LLINs, however, could not control *An arabiensis*, suggesting that additional targeted control tools should be tailored to reduce secondary vectors responsible for residual malaria transmission.

A Cochrane review reported that piperonyl-butoxide pyrethroid LLINs had a greater effect on malaria prevalence and mosquito population density than pyrethroid-only LLINs, in areas with high pyrethroid resistance.^3^, ^8^, ^9^, ^10^ A cluster randomised control trial (cRCT) in Tanzania and Benin showed strong evidence for a reduction of malaria prevalence and vector density in the chlorfenapyr pyrethroid LLINs compared with standard pyrethroid LLINs.^4^, ^11^ In addition, pyriproxyfen pyrethroid LLINs (an insect growth regulator) were more effective than pyrethroid LLINs against clinical malaria and highly pyrethroid-resistant vectors in a cRCT in Burkina Faso.^5^ However the previous cRCT did not explore differential effects on vectors species distribution and population density,^3^, ^4^, ^5^ which will be essential to tailor the most appropriate vector-control interventions.

In this Article, we detailed a secondary analysis from a large cRCT in Tanzania. The primary outcomes reporting the effectiveness of the dual active-ingredient LLINs on malaria indicators and overall vector density over 2 years follow-up have already been published.^4^ Here we are expanding the analysis by looking at the differential impact of those dual active-ingredient LLINs on the two main malaria-vector complexes found in the area, *An gambiae* sl and *An funestus* sl. In addition, we also report on the effects of dual active-ingredient LLINs on entomological outcomes during the third year of follow-up. Finally, we complement the entomological indicators by using an ecological niche model to investigate potential shifts in malaria-vector species distribution following the introduction of these interventions.

## Methods

### Study area and design

In this secondary analysis, entomological surveillance was done in 84 clusters formed from 72 villages, as part of a 3 year, single-blind, four parallel arm cRCT in the Misungwi district of north-western Tanzania.^4^ In the study area, the dominant malaria vector species were *An arabiensis* and *An funestus*, with *An funestus* being the leading malaria-vector species responsible for more than 90% of ongoing malaria transmission.^12^ Preintervention (August to December, 2018) pyrethroid resistance in malaria vectors in the study site was high (mean mortality <60%) after exposure to α cypermethrin or permethrin in standard insecticide-resistance bioassays.^12^ The main vector-control strategy is continuous distribution of LLINs to primary school students and during routine antenatal care visits to pregnant women. The study interventions were allocated to clusters (21 clusters per intervention) using a restricted randomisation approach. The following interventions were deployed: chlorfenapyr pyrethroid LLIN, containing a mixture of chlorfenapyr (4·8 g/kg) and α cypermethrin (2·4 g/kg; Interceptor G2, BASF SE, Ludwigshafen, Germany); piperonyl-butoxide pyrethroid LLIN, containing a mixture of piperonyl butoxide (10 g/kg) and permethrin (20 g/kg; Olyset Plus, Sumitomo Chemical, Tokyo, Japan); pyriproxyfen pyrethroid LLINs, containing a mixture of pyriproxyfen (5·5 g/kg) and α cypermethrin (5·5 g/kg; Royal Guard, Disease Control Technologies, Greer, SC, USA); and standard pyrethroid LLINs (reference group), containing α cypermethrin (5 g/kg; Interceptor). A detailed description of the study area and trial design has been reported previously.^12^, ^13^

### Procedures

**Figure 1:**
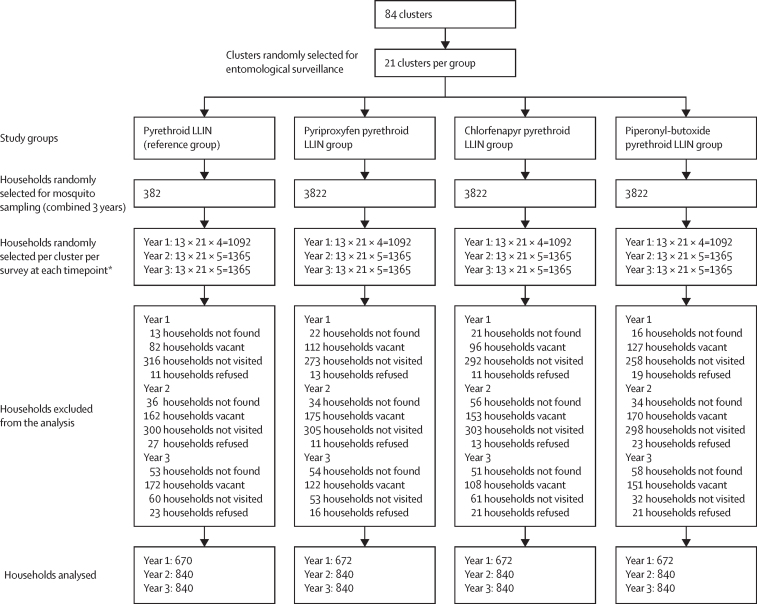
Trial profile For each cluster, 13 households (eight households and five extra households) were randomly selected for each survey collection round. The extra five households were visited only if one of the first eight houses was found vacant, the inhabitants refused further study, the household was not found, or the household was not visited because of a bad road. Four consecutive collection rounds in year 1 and five collection rounds in year 2 were done. A fifth round of collections was added in year 2 and year 3 to compensate time when collections were suspended (March and May) because of the SARS-CoV-2 pandemic. LLIN=long-lasting insecticidal net.

Study LLINs were distributed in January, 2019. 13 households were selected for entomological monitoring, eight main households and five additional households were randomly selected from the core area of each cluster from a census list generated during baseline enumeration. At the beginning of the household visit, we sought consent from the household heads or adult members of each household. Adult mosquitoes were collected quarterly using Centers for Disease Control and Prevention (CDC) light traps (John W Hock Company, Gainesville, FL, USA). CDC light traps were installed in a room, preferably with a single sleeping place, at the foot end, and the net found was replaced with a new standard Interceptor LLIN. Household information including net coverage and information related to household characteristics such as roofing and wall materials, type of eaves, and total number of residences were collected and recorded in an electronic form in Open Data Kit. Because of the SARS-CoV-2 pandemic, mosquito collections were suspended between March and May, 2020, thus one extra survey was carried out in year 2 and year 3 after intervention, to compensate. We provided an outline of the study design (figure 1).

Wild-collected adult female malaria vectors were morphologically identified, separating *Anopheles* from other mosquito genera, and then separated to species level, separating *An funestus* sensu lato sl from *An gambiae* complex. Up to ten *An funestus* sl and ten *An gambiae* sl per house collection were randomly selected for *Plasmodium falciparum* sporozoite detection using circumsporozoite-protein ELISA.^14^ DNA was extracted from the legs and wings and PCR TaqMan assays were done on all circumsporozoite-positive samples and a subset of specimens morphologically identified *An funestus* sl or *An gambiae* sl per cluster, to distinguish sibling species in *An gambiae*^15^ or *An funestus* complexes.^16^

### Outcomes

The primary outcomes were as follows: density of different malaria-vector species measured as the number of female *Anopheles* collected per household per night; entomological inoculation rate (EIR), an indicator of malaria transmission, defined as the average number of sporozoite-positive mosquitoes per night per household and weighted to account for the proportion of collected *Anopheles* to be processed for sporozoite analysis; and sporozoite rate, defined as the proportion of *Anopheles* found infected with malaria parasites. All entomological endpoints were analysed at 12 months, 24 months, and 36 months after distribution and compared between the interventional group and reference group. Secondary outcome was vector-species composition. Preintervention species-distribution models were used to assess changes after intervention in the geographical distribution of major malaria-vector species in each intervention group. We also did exploratory secondary analyses to assess the effect of the interventions on vector density at two geographical locations (northern and southern clusters) of the study area with varying epidemiological and ecological characteristics.

### Statistical analysis

The trial was designed to provide adequate power for the primary analysis. In this Article, the secondary analysis followed a preplanned analytical plan on the basis of a published protocol (trial registration number NCT03554616).^13^ Sample size was calculated for the main entomological outcome (EIR for all vector species combined) and detailed previously.^13^ Data were analysed regardless of whether the household members used the allocated study net (intention-to-treat analysis). Mixed-effects negative binomial regression analysis was used to estimate density ratios of the effect of each dual active-ingredient LLIN compared with the standard pyrethroid LLIN (reference group) on *An funestus*, *An gambiae* sl, or *Culex* species density, and EIR at different study points. The relative proportion of *Anopheles* species between study groups and study period were modelled using logistic regression. Sporozoite rates were compared between groups using mixed-effect logistic regression. The models accounted for clustering effect and were adjusted for baseline malaria prevalence, net usage, and vector density. The intervention groups, year, and their interaction, as well as baseline covariates, were fitted in the model as fixed effects, with collection round and cluster as random effects. The interaction was added to estimate time-specific effects of the individual interventions by reference to the standard pyrethroid LLIN during the 3 survey years after intervention. Average *Anopheles* caught per night per household, density ratios, and 95% CIs were presented. The same analysis was also done for the northern and southern clusters separately. All statistical analysis was done using Stata version 15.1. As the study net usage was low in the third year, a post-hoc per-protocol analysis was done for vector density and included only households that actually used the allocated study nets in the room in which the CDC light traps were installed.

### Ecological niche model analysis

To complement entomological collections, species-distribution models were initially constructed and validated before the randomisation of study clusters to intervention groups, to predict the geographical extent of different malaria-vector species across the study area.^13^ This approach served to reduce the potential imbalance between study groups caused by differences in vector-species composition across clusters. To this end, habitat suitability for each *Anopheles* species was determined using an ecological niche modelling approach, on the basis of a maximum entropy (MaxEnt version 3.4.1) algorithm.^17^ MaxEnt uses presence-only occurrence data in conjunction with environmental data to predict the potential distribution of a species across a defined landscape. In this study, the preintervention species-distribution models were used to assess changes after intervention in the geographical distribution of major malaria-vector species in each LLIN intervention group. This approach aimed to identify whether any observed differences in vector density between groups could be associated with geographical shifts in species habitat suitability, in addition to the direct effects of LLIN interventions on vector populations.

A pilot entomological survey was done in May, 2018, before the start of the cRCT. A total of 202 households across 92 villages were sampled for one night each using a CDC light trap. Separate niche models were constructed for *An arabiensis* and *An funestus* sl using pilot survey-occurrence records (n=69 for *An arabiensis* and n=51 for *An funestus* sl). Bioclimatic variables for model development at a 1 km resolution were obtained from the WorldClim database, including temperature seasonality, annual precipitation, precipitation of wettest month, precipitation of driest month, precipitation seasonality, and elevation. These variables were selected for their association with malaria-vector species in Tanzania.^17^ Data were randomly partitioned for model evaluation, with 75% of the records used as training data to construct the models and the remaining 25% set aside for testing. The accuracy of each model was determined by doing both a threshold-dependent binomial test of omission and a threshold-independent receiver-operating-characteristic analysis. Models for both species had high accuracy with area under the curve (AUC) of 0·82 for *An arabiensis* and AUC of 0·90 for *An funestus*. Species-specific models were further validated using independently-collected occurrence data from baseline entomological monitoring in all study clusters between August and November, 2018 (n=141 for *An funestus* and n=79 for *An arabiensis*); models had high external validity using the baseline occurrence data as test data (AUC=0·79 for *An funestus* and AUC=0·80 for *An arabiensis*).

Species-occurrence data from the 2 years following LLIN distribution were used as test records for external validation of preintervention niche models to assess changes after intervention in the geographical distribution of major malaria-vector species in the different study groups. The test AUC value was compared with initial model accuracy metrics obtained using baseline test data, whereby no change in AUC values indicated stability in species occurrence (ie, minimal change in species distribution after intervention relative to the preintervention period), whereas a decrease in AUC values indicated a shift or contraction in species occurrence after intervention relative to the predicted preintervention niche. A threshold was then applied to dichotomise the predicted habitat suitability into presence or absence and the test omission rate was calculated for each model.

### Role of the funding source

The funders of the study had no role in study design, data collection, data analysis, data interpretation, or writing of the report.

## Results

At baseline, household characteristics, LLIN coverage, and entomological and epidemiological indicators were similar in the four study groups.^4^ The predominant malaria vector was *An funestus* ss, accounting for 4700 (95%) of 4973 *Anopheles* vectors collected, and was responsible for more than 90% of malaria transmission in the study area.^12^ In January, 2019, 147 230 LLINs were distributed across study groups.^4^ A total of 14 entomological cross-sectional surveys were carried out between Feb 18, 2019, and Dec 6, 2021. A total of 15 288 houses were selected (13 per cluster per survey round) for entomological collections; 9403 (62%) households consented to participate. The remaining households were found vacant during the survey period (1630 [11%]), households that we could not reach due to heavy rain (343 [2%]), households that were not found because they either moved or relocated (448 [3%]), and a small proportion of households that refused to participate in the study (209 [1%]). 3255 (21%) households were not visited because we had met our sample size of eight consenting households per cluster (figure 1).

A total of 146 936 mosquitoes were collected over the 3 successive years, of which 34 605 (23·6%) were female anophelines, principally *An funestus* sl and *An gambiae* sl. 110 246 samples were culicines and the remaining were other mosquitoes, including male mosquitoes.

We observed differential effects of the dual active-ingredient LLINs on separate vector species, with the greatest effect on *An funestus*. Over the full trial period, mean *An funestus* sl density was 3∙1 per house per night in the standard pyrethroid LLIN group and significantly lower in the chlorfenapyr pyrethroid LLIN group (mean density 1∙2 per house per night). There was consistently significantly lower densities of *An funestus* sampled in the chlorfenapyr pyrethroid LLIN group than in the standard pyrethroid LLIN group at year 1, at year 2, and at year 3 (table 1) after intervention. Similarly, overall *An funestus* density was 1·4 per household in the piperonyl-butoxide pyrethroid LLIN group, compared with 3·1 in the standard pyrethroid LLIN group (reference group). The strongest effect of piperonyl-butoxide pyrethroid LLINs against *An funestus* density was seen in year 1 and year 2, with weak evidence in year 3 after net distribution.

**Table 1:** Differential effects of the dual LLINs compared with standard pyrethroid LLIN on *Anopheles* species 12 months, 24 months, and 36 months after intervention

	**Number of households analysed**	**Total *Anopheles funestus* sl**	**Density per night per household**	**aDR**	**95% CI**	**p value**	**Total *Anopheles gambiae* sl**	**Density per night per household**	**aDR**	**95% CI**	p value*
**Year 1: 2019**											
Pyrethroid-only LLIN group (reference)	670	1355	2·0	1	..	..	303	0·5	1	..	..
Chlorfenapyr pyrethroid LLIN group	671	118	0·2	0·14	0·07–0·28	<0·0001	317	0·5	0·81	0·39–1·70	0·57
Piperonyl-butoxide pyrethroid LLIN group	672	276	0·4	0·33	0·17–0·65	0·0014	224	0·3	0·58	0·27–1·22	0·15
Pyriproxyfen pyrethroid LLIN group	672	527	0·8	0·56	0·29–1·08	0·082	268	0·4	0·74	0·36–1·56	0·43
**Year 2: 2020**											
Pyrethroid-only LLIN group (reference)	838	4323	5·2	1	..	..	2029	2·4	1	..	..
Chlorfenapyr pyrethroid LLIN group	840	2040	2·4	0·32	0·18–0·58	<0·0001	3018	3·6	0·88	0·47–1·67	0·71
Piperonyl-butoxide pyrethroid LLIN group	840	1751	2·1	0·48	0·27–0·85	0·011	2163	2·6	1·15	0·61–2·15	0·66
Pyriproxyfen pyrethroid LLIN group	840	5041	6·0	0·86	0·49–1·53	0·61	2592	3·1	1·14	0·61–2·14	0·68
**Year 3: 2021**											
Pyrethroid-only LLIN group (reference)	840	1626	1·9	1	..	..	657	0·8	1	..	..
Chlorfenapyr pyrethroid LLIN group	840	740	0·9	0·31	0·17–0·56	<0·0001	948	1·1	0·80	0·42–1·54	0·51
Piperonyl-butoxide pyrethroid LLIN group	840	1294	1·5	0·66	0·37–1·18	0·16	470	0·6	0·56	0·29–1·08	0·086
Pyriproxyfen pyrethroid LLIN group	840	1572	1·9	0·72	0·40–1·29	0·27	953	1·1	0·77	0·40–1·48	0·43
**Overall (3 years combined)**											
Pyrethroid-only LLIN group (reference)	2348	7304	3·1	1	..	..	2989	1·3	1	..	..
Chlorfenapyr pyrethroid LLIN group	2351	2898	1·2	0·26	0·17–0·41	<0·0001	4283	1·8	0·83	0·55–1·25	0·37
Piperonyl-butoxide pyrethroid LLIN group	2352	3321	1·4	0·49	0·32–0·76	0·0012	2857	1·2	0·75	0·50–1·12	0·16
Pyriproxyfen pyrethroid LLIN group	2352	7140	3·0	0·72	0·47–1·12	0·15	3813	1·6	0·89	0·60–1·34	0·58

The intervention group was compared to the standard pyrethroid LLIN group at each timepoint. Density ratios were adjusted for baseline cluster-level variables used in restricted randomisation. We applied a Bonferroni correction for multiplicity given the multiple comparison groups. p<0·017 was considered statistically significant. LLIN=long-lasting insecticidal net. aDR=adjusted density ratio.

There was weak evidence of a difference in *An funestus* population density in the pyriproxyfen pyrethroid LLIN group, compared with the standard pyrethroid LLIN group. There was variation in indoor *An funestus* population density across the survey period (appendix p 3). None of the dual active-ingredient LLINs were effective in reducing the density of *An arabiensis* (table 1). Per-protocol analysis gave similar results (appendix p 4).

There was no strong evidence in any of the interventions groups of reduced overall sporozoite rate, compared with the standard pyrethroid LLIN group, over the 3 years of the trial (table 2). Overall, there were reductions in malaria transmission, with *An funestus* EIR (per night per household) being significantly lower in the chlorfenapyr pyrethroid LLINs group than in the standard pyrethroid LLINs group throughout the study period (table 2). EIR per night per household combined over 3 years was 0·03 in the piperonyl-butoxide pyrethroid LLINs group compared with 0·06 in the standard pyrethroid LLINs group (table 2). The EIRs were not statistically different between groups that received pyriproxyfen pyrethroid LLINs compared with pyrethroid LLINs at any time point (table 2). Only chlorfenapyr pyrethroid LLINs had a significant effect on *An funestus* EIR at the third year of follow-up (table 2).

**Table 2:** Effect of dual LLINs compared with standard pyrethroid LLIN on sporozoite rate and EIR

	**Number of households analysed**	**Number positive**	**Sporozoite rate**	**Odds ratio**	**95% CI**	**p value**	**EIR per night per household**	**aDR**	**95% CI**	p value*
**Year 1**										
Pyrethroid-only LLIN group (reference)	724	14	1·9%	1	..	..	0·04	1	..	..
Chlorfenapyr pyrethroid LLIN group	113	2	1·8%	1·17	0·24–5·77	0·84	0·00	0·14	0·03–0·72	0·019
Piperonyl-butoxide pyrethroid LLIN group	250	4	1·6%	0·74	0·21–2·58	0·63	0·01	0·25	0·07–0·96	0·044
Pyriproxyfen pyrethroid LLIN group	388	11	2·8%	1·83	0·71–4·71	0·21	0·02	0·89	0·34–2·31	0·81
**Year 2**										
Pyrethroid-only LLIN group (reference)	1740	46	2·6%	1	..	..	0·09	1	..	..
Chlorfenapyr pyrethroid LLIN group	901	12	1·3%	0·59	0·27–1·28	0·18	0·02	0·18	0·06–0·44	<0·0002
Piperonyl-butoxide pyrethroid LLIN group	1046	32	3·1%	0·98	0·53–1·81	0·95	0·06	0·48	0·24–0·98	0·044
Pyriproxyfen pyrethroid LLIN group	1524	32	2·1%	0·92	0·50–1·70	0·81	0·10	0·67	0·29–1·55	0·35
**Year 3**										
Pyrethroid-only LLIN group (reference)	1184	28	2·4%	1	..	..	0·04	1	..	..
Chlorfenapyr pyrethroid LLIN group	591	11	1·9%	0·96	0·41–2·27	0·93	0·02	0·32	0·14–0·74	0·0079
Piperonyl-butoxide pyrethroid LLIN group	885	21	2·4%	0·97	0·47–2·01	0·93	0·03	0·67	0·27–1·67	0·39
Pyriproxyfen pyrethroid LLIN group	1118	13	1·2%	0·57	0·25–1·27	0·17	0·02	0·53	0·21–1·36	0·19
**Overall (3 years combined)**										
Pyrethroid-only LLIN group (reference)	3648	88	2·4%	1	..	..	0·06	1	..	..
Chlorfenapyr pyrethroid LLIN group	1605	25	1·6%	0·77	0·43–1·38	0·38	0·01	0·21	0·14–0·33	<0·0001
Piperonyl-butoxide pyrethroid LLIN group	2181	57	2·6%	0·95	0·59–1·55	0·85	0·03	0·49	0·31–0·79	0·0037
Pyriproxyfen pyrethroid LLIN group	3030	56	1·8%	0·90	0·55–1·47	0·68	0·05	0·66	0·37–1·18	0·16

The intervention group was compared with the standard pyrethroid LLIN group at each timepoint. Odds ratios for sporozoite rates and density ratios for EIR were adjusted for baseline cluster-level variables used in restricted randomisation. EIR aDRs were weighted to account for the proportion of mosquitoes sampled to be tested for sporozoites. We applied a Bonferroni correction for multiplicity given the multiple comparison group. p<0·017 was considered statistically significant. LLIN=long-lasting insecticidal net. EIR=entomological inoculation rate. aDR=adjusted density ratio.

Chlorfenapyr pyrethroid LLINs were also effective in reducing *Culex* density every year, with the strongest effect in year 1 and an average of 9·9 per night per house in the standard pyrethroid LLIN group and 6·2 in the chlorfenapyr pyrethroid LLIN group (adjusted density ratio 0·36, 0·20–0·66; p=0·0009). Overall, chlorfenapyr pyrethroid LLINs significantly reduced the density of all indoor mosquitoes by 54% (density ratio 0·46, 0·31–0·67; p<0·0001), compared with standard pyrethroid LLINs during 3 years of community use (appendix p 5). A decrease in *Culex* species density was also observed in the houses with piperonyl-butoxide pyrethroid LLINs and pyriproxyfen pyrethroid LLIN compared with standard pyrethroid LLINs, with the most important difference observed in the second year after distribution (appendix p 5–6).

In the study area, the relative vector-species abundance and distributions varied between southern and northern clusters (appendix p 7). Comparing the effects of the interventions in two locations of the study area, the dual active-ingredient LLINs had a greater effect in the southern areas than in the northern clusters. In the southern clusters, vector density was 70% lower (density ratio 0·30, 95% CI 0·19–0·50; p<0·0001) in chlorfenapyr pyrethroid LLIN clusters and 54% lower (density ratio 0·46, 0·27–0·80; p=0·0057) in piperonyl-butoxide pyrethroid LLIN clusters; there was no significant reduction in pyriproxyfen pyrethroid LLIN clusters compared with the standard LLIN clusters (appendix p 7). There was evidence of an effect on vector density in the northern clusters, with the strongest effect seen for *An funestus* in the chlorfenapyr pyrethroid LLIN clusters, and some limited effect in the piperonyl-butoxide pyrethroid LLIN clusters (appendix p 7), compared with pyrethroid LLIN clusters.

Most *An funestus* sl collected were *An funestus* ss (81·7%, 95% CI 75·7–86·5) across the four study groups. Other species found at lower proportions included *An rivulorium* (1·3%, 0·8–2·2) and *An parensis* (17·0%, 12·5–22·6). Of the *An gambiae* sl analysed to species level (n=2945), most individuals were *An arabiensis* (93·8%, 86·3–97·3); the remaining species were *An gambiae* ss (6·2%, 2·6–13·7). We reported *Anopheles* sibling-species composition per study group over 2 survey years (table 3; appendix p 8). There was a difference in the relative proportions of *An funestus* and *An arabiensis*, the two most common vectors, between study groups. In the chlorfenapyr pyrethroid LLIN group, there was an overall significant reduction in relative proportion of *An funestus*, mainly *An funestus* ss (40·4%, 31·8–48·9, *vs* 71·0%, 65·4–76·5; odds ratio [OR] 0·28, 95% CI 0·14–0·57; p<0·0001) compared with the standard pyrethroid LLIN group (table 3). This difference was observed in each follow-up year. Similar findings were observed in the piperonyl-butoxide pyrethroid LLIN group during the first year of follow-up (55·2%, 45·6–64·8, *vs* 81·7%, 76·3–87·1; OR=0·28, 0·12–0·64; p=0·0026) and we found borderline weak evidence of reductions of *An funestus* in the piperonyl-butoxide pyrethroid LLIN group relative to the standard pyrethroid LLIN group in the second year. A non-significant difference in species composition was also observed in the pyriproxyfen pyrethroid LLIN group compared with the standard pyrethroid LLIN group (table 3).

**Table 3:** Proportion of *Anopheles funestus* 1 year, 2 years, and 3 years after intervention and overall

	**Proportion of *Anopheles Funestus* (n/N)**	**Comparison between groups**			**Comparison between years**		
		Odds ratio	95% CI	p value	Odds ratio	95% CI	p value
**Year 1: 2019**							
Pyrethroid-only LLIN group (reference)	81·7% (1355/1658)	1 (ref)	..	..	Ref	..	..
Chlorfenapyr pyrethroid LLIN group	27·1% (118/435)	0·08	0·04–0·18	<0·0001	Ref	..	..
Piperonyl-butoxide pyrethroid LLIN group	55·2% (276/500)	0·28	0·12–0·64	0·0026	Ref	..	..
Pyriproxyfen pyrethroid LLIN group	66·3% (527/795)	0·44	0·18–1·05	0·065	Ref	..	..
**Year 2 : 2020**							
Pyrethroid-only LLIN group (reference)	68·1% (4323/6352)	1 (ref)	..	..	0·48	0·23–0·97	0·042
Chlorfenapyr pyrethroid LLIN group	40·3% (2040/5058)	0·32	0·13–0·80	0·015	1·82	0·81–4·07	0·15
Piperonyl-butoxide pyrethroid LLIN group	44·7% (1751/3914)	0·38	0·15–0·98	0·045	0·66	0·36–1·19	0·17
Pyriproxyfen pyrethroid LLIN group	66·0% (5041/7633)	0·91	0·31–2·68	0·87	0·99	0·51–1·90	0·97
**Year 3: 2022**							
Pyrethroid-only LLIN group (reference)	71·2% (1626/2283)	1 (ref)	..	..	0·55	0·28–1·09	0·087
Chlorfenapyr pyrethroid LLIN group	43·8% (740/1688)	0·32	0·16–0·63	0·0010	2·10	0·96–4·58	0·063
Piperonyl-butoxide pyrethroid LLIN group	73·3% (1294/1764)	1·11	0·59–2·08	0·74	2·23	1·11–4·48	0·024
Pyriproxyfen pyrethroid LLIN group	62·3% (1572/2525)	0·67	0·39–1·14	0·14	0·84	0·46–1·53	0·57
**Overall (3 years combined)**							
Pyrethroid-only LLIN group (reference)	71·0% (7304/10 293)	1 (ref)	..	..	..	..	..
Chlorfenapyr pyrethroid LLIN group	40·4% (2898/7181)	0·28	0·14–0·57	<0·0001	..	..	..
Piperonyl-butoxide pyrethroid LLIN group	53·8% (3321/6178)	0·48	0·24–0·94	0·033	..	..	..
Pyriproxyfen pyrethroid LLIN group	65·2% (7140/10 953)	0·78	0·34–1·81	0·57	..	..	..

Comparisons were done between each of the interventions groups versus the reference group (pyrethroid only) for each year separately, and then combined. The proportion of *An funestus* found in each group in year 2 and year 3 were compared with the result of the same group in year 1. The proportion of *An funestus* was calculated as n being the total number of female *An funestus* collected and N the total number of all female vectors collected (*An funestus* and *Anopheles gambiae* sl). We applied a Bonferroni correction for multiplicity given the multiple comparison groups. p<0·017 was considered statistically significant. LLIN=long-lasting insecticidal net.

**Figure 2:**
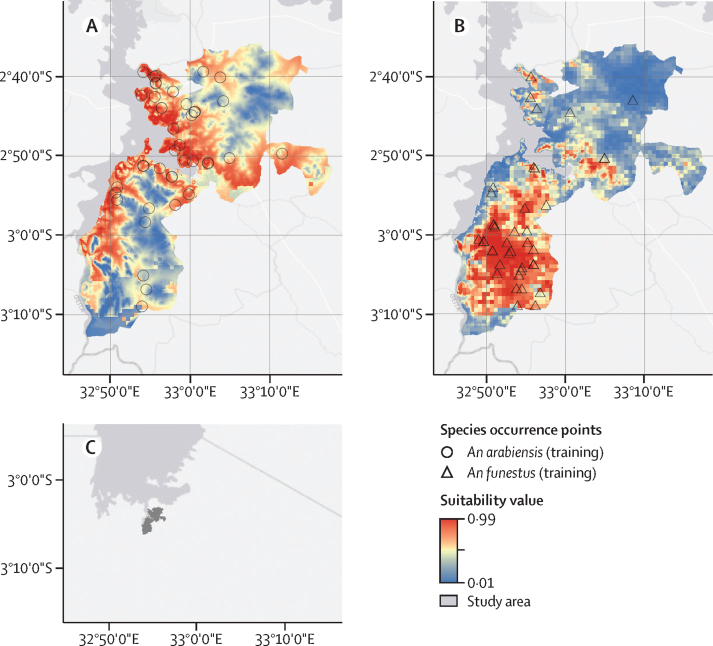
Species distribution Predicted species distribution maps based on ecological models for (A) *Anopheles arabiensis* and (B) *Anopheles funestus* in Misungwi district, Tanzania. The probabilistic habitat suitability values for each species range from 0 (not suitable) to 1 (highly suitable). Occurrence point locations used for MaxEnt model training are shown for each species. Maps were created using ArcGIS software by Esr. Basemap sources: Esri, HERE, Garmin, FAO, NOAA, USGS, OpenStreetMap contributors, and the GIS User Community.^36^

When tested with independent postintervention data, MaxEnt pilot ecological niche models for *An funestus* and *An arabiensis* had robust evaluation metrics (appendix p 9). The test omission rate did not differ markedly between timepoints, indicating that ecological niches for both *An funestus* and *An arabiensis* were quite stable over time (ie, no major observed shift in species geographical range; figure 2).

## Discussion

In rural Tanzania, where malaria is transmitted by highly pyrethroid-resistant vectors, dual active-ingredient LLINs, incorporating chlorfenapyr or piperonyl butoxide, were more effective than standard pyrethroid LLINs. The impact of dual active-ingredient LLINs was more evident on the major malaria vector species *An funestus* than on *An arabiensis*. In the present trial, the improved effect of two of the dual active-ingredient LLINs on entomological outcomes was supported by significant reductions in malaria prevalence by 55% in the chlorfenapyr pyrethroid LLIN group over the first 2 years of study and by 47% in the piperonyl-butoxide pyrethroid LLIN group during year 1 of the trial.^4^ The differential effects of the dual active-ingredient LLINs on malaria vector species provides some evidence about the importance of using tailored interventions based on the local context.

In clusters provided with chlorfenapyr pyrethroid LLINs, *An funestus* population density was significantly reduced by 74%, resulting in a 79% reduction in EIR, compared with clusters with standard pyrethroid LLINs over 3 years of community use. Despite a high decline in chlorfenapyr pyrethroid LLIN usage over time (68% at 12 months and 23% at 36 months) and close to 80% loss in chlorfenapyr content after 24 months,^4^ the nets remained highly effective against *An funestus*, reducing density and EIR up to the third year of follow-up, which is probably because of *An funestus* being susceptible to chlorfenapyr even at low dosage or carry-over effects from the net use in previous years. Previous small-scale semi-field experimental hut trials in Tanzania showed consistently higher mortality against *Anopheles gambiae* ss and *An funestus* in chlorfenapyr pyrethroid LLINs compared with pyrethroid-only LLINs, demonstrating no loss of efficacy of the chlorfenapyr component in the nets even after being washed 20 times.^18^ Similarly, chlorfenapyr pyrethroid LLIN efficacy was sustained after 20 washes, causing high mortality similar to unwashed nets against pyrethroid-resistant *An coluzzii*, *An gambiae* ss, and *An arabiensis* in west Africa.^19^, ^20^, ^21^

Community acceptance of interventions relies on reductions in biting nuisance from all mosquitoes, in addition to an effect on malaria transmission. In the present trial, the chlorfenapyr pyrethroid LLINs were effective in reducing the biting nuisance of non-malaria mosquitoes such as *Culex* species and other *Anopheles*. In this study, female *Culex* species contributed to 69% of the indoor mosquito population density. Overall, there was a statistically significant decrease in *Culex* species density by 48% and all mosquitoes by 54% in clusters given chlorfenapyr pyrethroid LLINs compared with pyrethroid LLINs. Previous hut trials have demonstrated the effectiveness of chlorfenapyr on *Culex* species,^22^ suggesting its potential against other vector-borne diseases, in line with WHO Integrated Vector Management strategies.

Overall, piperonyl-butoxide pyrethroid LLINs were more effective than standard pyrethroid LLINs and reduced *An funestus* density by 51%, with the strongest effect (67% reduction) observed in year 1 and year 2; this reduction declined over time and was not sustained up to the third year after intervention. The shorter effect of piperonyl-butoxide pyrethroid LLINs is inconsistent with previous studies in which the intervention effectiveness was sustained for 2 years in Tanzania^8^, ^23^ and in Uganda.^9^, ^10^ Piperonyl-butoxide pyrethroid LLINs could be more efficacious against *An gambiae* ss, the main malaria vector found in these two trials,^8^, ^23^, ^24^ unlike in Misungwi where *An funestus* predominated.^4^, ^12^ *An gambiae* ss metabolic resistance is mediated by overexpression of cytochrome P450, whereas resistance mechanisms other than those that are P450 based,^25^ such as cuticle thickening, could also be involved in *An funestus* resistance.^26^ Other possible reasons for the reduced efficacy of piperonyl-butoxide pyrethroid LLINs could be poor durability or rapid depletion of the piperonyl-butoxide content on the surface of the nets, resulting in loss of the killing effect against resistant vectors, which could affect community usage of theses nets.^4^ In the Ugandan cRCT, the LLIN durability study found that piperonyl-butoxide pyrethroid LLINs lost bioefficacy and was associated with a decline in piperonyl-butoxide content after 2 years of community use.^27^ However, bioefficacy could vary between different brands of LLINs and study locations that require additional evidence. In our trial, although EIRs maintained by *An funestus* were lower in the piperonyl-butoxide pyrethroid LLIN group, compared with the standard pyrethroid LLIN group over the study period, these reductions were not statistically different.

There was some indication that pyriproxyfen pyrethroid LLINs reduced the *An funestus* population density compared with standard-pyrethroid LLINs in year 1, however the effect was not statistically significant; this finding is in contrast to a 1 year cRCT done in Burkina Faso in which pyriproxyfen pyrethroid LLINs reduced vector density by 22% and EIR by 51% compared with standard LLINs.^5^ In the Burkina Faso cRCT, despite almost halving the EIR, the overall reduction in incidence of clinical malaria was quite low, 12% in the pyriproxyfen pyrethroid LLIN group. In our current study, the decline in net usage over the study, loss of pyriproxyfen bioefficacy,^4^ and possible pyriproxyfen resistance in the main malaria vector *An funestus* could explain the low effectiveness of this LLIN in the study setting. Yunta and colleagues^28^ in 2016 reported that pyriproxyfen can be metabolised by cytochrome P450s, enzymes associated with pyrethroid resistance in *Anopheles*, thereby reducing the bioefficacy of pyriproxyfen pyrethroid LLINs. In addition to inducing vector mortality, pyriproxyfen pyrethroid LLINs have a sterilising effect in female mosquitoes;^29^, ^30^ however, in the current study, a small proportion (127 [24%] of 536) of *An funestus* mosquitoes were sterilised.^4^ Combining pyriproxyfen and a pyrethroid might be antagonistic because of the excitorepellent effect of the pyrethroid, thus reducing vector intervention contact time, leading to less sterilisation of pyriproxyfen in mosquitoes. In the present study, there was some evidence that pyriproxyfen pyrethroid LLINs could reduce the density of non-malaria vectors including male mosquitoes, by 37%, compared with the standard pyrethroid LLINs group.

The absence of an impact of indoor interventions on *An arabiensis* has also been described in previous studies in which standard pyrethroid LLINs did not control this species,^31^ probably because of their outdoor biting and resting behaviour. In the present study, the relative proportion of *An funestus* within all malaria vectors was 40·4% in the chlorfenapyr pyrethroid LLIN group after 3 years, corresponding to a 72% significant decrease when compared with the standard pyrethroid LLIN group; although relative proportions of *An arabiensis* significantly increased by four times in the chlorfenapyr pyrethroid LLIN group. It is likely that *An arabiensis* can maintain residual transmission especially when there is a shift in sibling-species composition and elimination of primary malaria vectors *An funestus*, *An gambiae* ss, or both by indoor interventions, as reported previously;^32^ thus, supplementary outdoor interventions are urgently needed in our study area.^33^, ^34^ There was no observed change in species habitat suitability over the postintervention period that might have affected observed species density, suggesting that the density changes were caused by differential intervention effects on vector species and no other environmental factors. This finding raises concerns of the potential for *An funestus* to rebound if high coverage of chlorfenapyr pyrethroid LLINs is removed. It also highlights the importance of using ecological modelling to identify geographical differences in vector-species distributions that can be used to inform cRCT designs, and to guide the deployment of different targeted interventions to improve the effectiveness of malaria control in areas with diverse vector species.

Consistent with baseline entomological findings and the ecological niche, we observed microgeographical differences in species composition and density of *Anopheles* vector mosquitoes in the study area, with higher vector densities in the southern clusters than in the northern clusters;^12^ *An funestus* was the most abundant vector in the southern part of the study area, whereas *An arabiensis* was uniformly distributed across both north and south clusters,^12^ consistent with the predicted preintervention species distributions. In the study area, *An funestus* was the leading and most important malaria vector, responsible for more than 90% of malaria transmission, because of its anthropophagical and endophagical behaviour.^12^ When comparing the effect of interventions between the two portions of the study area, we found that the chlorfenapyr pyrethroid LLINs reduced vector density by 70% in high-transmission settings in the south and by 40% in the northern parts of the study area, owing largely to differences in species composition. Similarly, piperonyl-butoxide pyrethroid LLINs had a greater effect in the southern clusters than northern clusters, suggesting vector species-specific control could be targeted in high-transmission settings, to maximise the impact of these interventions against malaria transmission. Previous studies support the effect of the spatial targeted-vector control on malaria transmission.^35^ A limitation of this study is the sharp decrease in study-net usage that might explain the reduced effect size between the three dual active-ingredient LLINs groups and the control group over time.

In conclusion, this study confirmed the superiority of two dual active-ingredient LLINs, containing either chlorfenapyr or piperonyl butoxide, compared with standard pyrethroid LLINs, against the major malaria vector species, *An funestus*, with chlorfenapyr pyrethroid LLINs being the most effective over 3 years of community use. Dual active-ingredient LLINs were not effective in controlling *An arabiensis*. The highest effect was observed in the southern part of the study area, with recommendations for targeted species-specific control in high-burden areas to sustain gains in malaria control.

## Data sharing

The datasets generated or analysed during this study are presented within the Article and its supporting appendix.

## Declaration of interests

We declare no competing interests.
