# Supplementary material for: Differential impact of dual-active ingredient long-lasting insecticidal nets on primary malaria vectors: a secondary analysis of a 3-year, single-blind, cluster-randomised controlled trial in rural Tanzania
Source: Lancet Planet Health. 2023 May 8;7(5):e370–80. doi: 10.1016/S2542-5196(23)00048-7 (PMC10186178; doi:10.1016/S2542-5196(23)00048-7)
Supplement: Supplementary appendix [file mmc1.pdf]

THE LANCET
Planetary Health

Supplementary appendix

This appendix formed part of the original submission and has been peer reviewed. We post it as supplied by the authors.

Supplement to: Matowo NS, Kulkarni MA, Messenger LA, et al. Differential impact of dual-active ingredient long-lasting insecticidal nets on primary malaria vectors: a secondary analysis of a 3-year, single-blind, cluster-randomised controlled trial in rural Tanzania. *Lancet Planet Health* 2023; **7:** e370–80

Differential impact of dual-active ingredient long-lasting insecticidal nets (LLINs) on primary malaria vectors: a secondary analysis of a three-year cluster-randomized controlled trial in rural Tanzania

Nancy S. Matowo PhD, Manisha A. Kulkarni PhD, Louisa A. Messenger PhD, Mohamed Jumanne BSc, Jackline Martin MSc, Elizabeth Mallya BSc, Eliud Lukole MSc, Jacklin F. Mosha PhD, Oliva Moshi BSc, Boniface Shirima BSc, Robert Kaaya MSc, Professor Mark Rowland PhD, Alphaxard Manjurano PhD, Professor Franklin W Mosha PhD, Natacha Protopopoff PhD

SUPPLEMENTARY APPENDIX

Contents

S1: Trends in female Anopheles species density distribution over time across the study arm 3

S2: Per protocol analysis..4

S3: Effect of dual-LLINs on non-malaria vectors...5

S4: Trends in female Culex species density distribution over time across the study arm....................6

S5: Spatial differences of effectiveness of dual-AI LLINs..7

S6: Anopheles species composition 8

S7: Maxent model 9

S1: Trends in female Anopheles species density distribution over time across the study arm

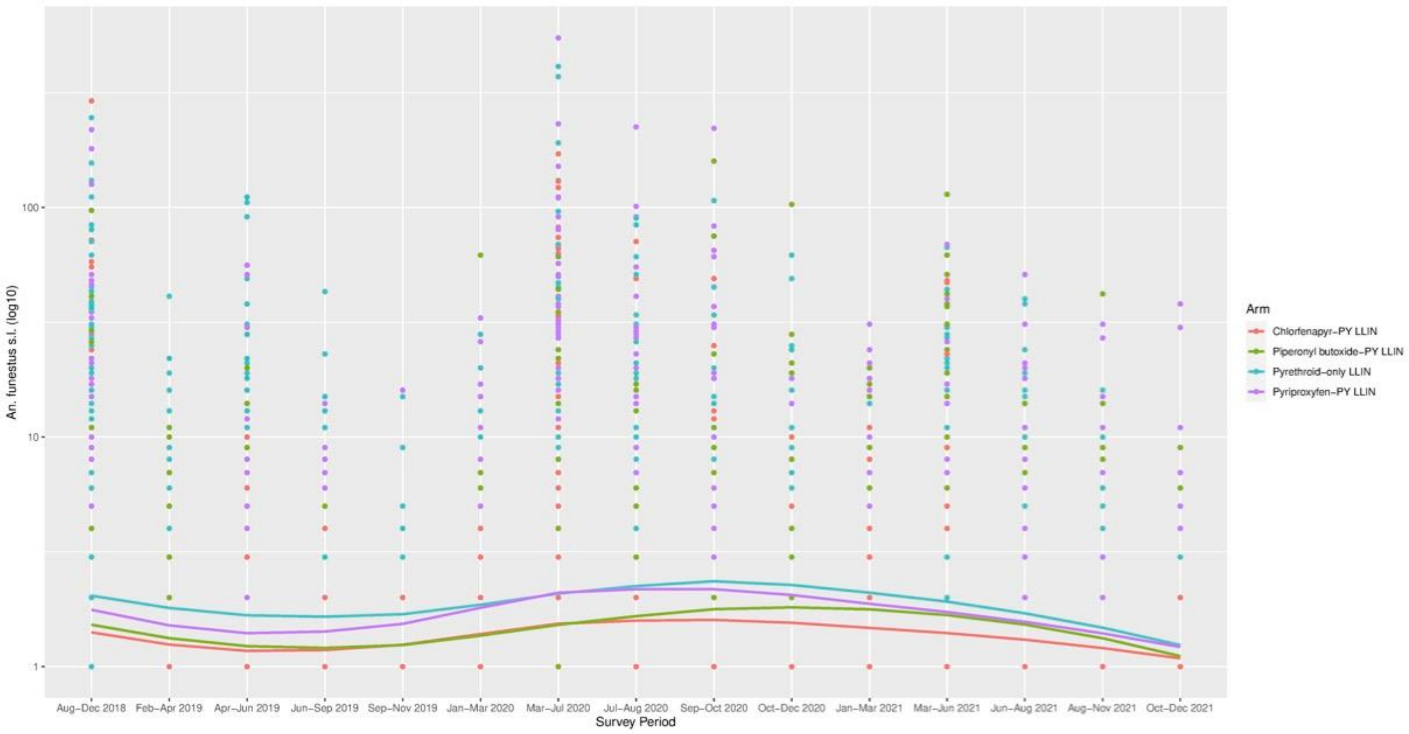

Trends of *An. funestus* population density distribution with smoothed averages over time across the treatment allocation arms defined as the orange (Chlorfenapyr-PY LLIN), green (Piperonyl butoxide-PY LLIN), blue (Pyriproxyfen-PY LLIN), and purple (Standard-Pyrethroid LLIN) with their respective 95% confidence intervals portrayed as grey shading. The plots were generated in R version 4.1.3 (R Development Core Team) using the "ggplot" packages.

S2: Per protocol analysis

Table: Post hoc per protocol analysis showing differential effects of the dual-LLINs compared to standard-PY LLIN on Anopheles species at 12, 24, 36 months, and combined after intervention

	Number of households analysed	*Total Anopheles funestus s.l.*	Density / night/ HH	DR	95%CI	p value*	*Total Anopheles gambiae s.l*	Density / night/ HH	DR	95%CI	p value*
**Year 1: 2019**											
Pyrethroid-only LLIN arm (ref)	511	1131	2.2	1			247	0.5	1		
Chlorfenapyr-PY LLIN arm	519	102	0.2	0.17	0.08-0.34	<0.0001	229	0.4	0.78	0.37-1.65	0.5172
Piperonyl butoxide-PY LLIN arm	510	228	0.4	0.40	0.20-0.80	0.0088	184	0.4	0.63	0.30-1.34	0.2287
Pyriproxyfen-PY LLIN arm	525	473	0.9	0.65	0.34-1.27	0.2062	216	0.4	0.73	0.35-1.55	0.4170
**Year 2: 2020**											
Pyrethroid-only LLIN arm (ref)	596	3309	5.6	1			1743	2.9	1		
Chlorfenapyr-PY LLIN arm	574	1519	2.6	0.28	0.16-0.51	<0.0001	2520	4.4	0.99	0.52-1.88	0.9767
Piperonyl butoxide-PY LLIN arm	458	938	2.0	0.48	0.27-0.87	0.0151	1374	3.0	1.13	0.60-2.17	0.6959
Pyriproxyfen-PY LLIN arm	520	3619	7.0	0.76	0.42-1.35	0.3450	1634	3.1	1.07	0.56-2.04	0.8325
**Year 3: 2021**											
Pyrethroid-only LLIN arm (ref)	430	1007	2.3	1			403	0.9	1		
Chlorfenapyr-PY LLIN arm	425	433	1.0	0.30	0.16-0.55	<0.0001	542	1.3	0.86	0.43-1.72	0.6649
Piperonyl butoxide-PY LLIN arm	235	502	2.1	0.62	0.31-1.21	0.1598	121	0.5	0.62	0.28-1.35	0.2251
Pyriproxyfen-PY LLIN arm	320	809	2.5	0.72	0.38-1.36	0.3069	418	1.3	0.85	0.41-1.77	0.6650
**Overall (3 years combined)**											
Pyrethroid-only LLIN arm (ref)	1537	5447	3.5	1			2393	1.6	1		
Chlorfenapyr-PY LLIN arm	1518	2054	1.4	0.25	0.16-0.39	<0.0001	3291	2.2	0.88	0.57-1.34	0.6464
Piperonyl butoxide-PY LLIN arm	1203	1668	1.4	0.50	0.32-0.77	0.0019	1679	1.4	0.82	0.53-1.27	0.3738
Pyriproxyfen-PY LLIN arm	1365	4901	3.6	0.72	0.47-1.11	0.1357	2268	1.7	0.90	0.59-1.39	0.5369

The intervention arm is compared to the standard-PY LLIN arm at each time point. LLIN=long-lasting insecticidal net. PY=Pyrethroid. DR=Density reduction ratio. DRs are adjusted for baseline cluster-level variables used in restricted randomization. *EIR are weighted to account for the proportion of mosquitoes sampled to be tested for sporozoites. We have applied a Bonferroni correction for multiplicity given the multiple comparison arms and *a p value <0·017 was considered statistically significant.

S3: Effect of dual-LLINs on non-malaria vectors

Table: Effect of dual-LLINs compared with the standard-PY LLIN on non-malaria vectors

	Number of households analysed	Female *Culex species* collected	Density / night/ HH	DR	95%CI	p value*	Total all mosquito collected	Density / night/ HH	DR	95%CI	p value*
**Year 1: 2019**											
Pyrethroid-only LLIN arm (ref)	670	6630	9.9	1			9100	13.6	1		
Chlorfenapyr-PY LLIN arm	671	3741	6.2	0.36	0.20-0.66	0.0009	4937	7.4	0.36	0.22-0.61	0.0001
Piperonyl butoxide-PY LLIN arm	672	3820	5.7	0.66	0.36-1.19	0.1634	4738	7.1	0.56	0.34-0.92	0.0237
Pyriproxyfen-PY LLIN arm	672	3741	5.6	0.53	0.29-0.96	0.0359	5378	8.0	0.55	0.33-0.91	0.0206
**Year 2: 2020**											
Pyrethroid-only LLIN arm (ref)	838	13909	16.6	1			21640	25.8	1		
Chlorfenapyr-PY LLIN arm	840	15541	18.5	0.58	0.33-1.01	0.0535	22162	26.4	0.51	0.32-0.82	0.0053
Piperonyl butoxide-PY LLIN arm	840	8780	10.5	0.50	0.29-0.85	0.0115	13421	16.0	0.52	0.32-0.83	0.0058
Pyriproxyfen-PY LLIN arm	840	12639	15.0	0.50	0.29-0.86	0.0128	21553	25.7	0.59	0.37-0.95	0.0283
**Year 3: 2021**											
Pyrethroid-only arm LLIN (ref)	840	10027	11.9	1			13431	16.0	1		
Chlorfenapyr-PY LLIN arm	840	7941	9.5	0.60	0.34-1.05	0.0748	10653	12.7	0.49	0.30-0.79	0.0036
Piperonyl butoxide-PY LLIN arm	840	5773	6.9	0.72	0.42-1.26	0.2516	8226	9.8	0.60	0.38-0.96	0.0341
Pyriproxyfen-PY LLIN arm	840	8312	9.9	0.94	0.54-1.64	0.8395	11697	13.9	0.74	0.46-1.19	0.2172
**Overall (all three years combined)**											
Pyrethroid-only LLIN arm (ref)	2348	30566	13.0	1			44171	18.8	1		
Chlorfenapyr-PY LLIN arm	2351	27627	11.8	0.52	0.33-0.81	0.0037	37752	16.1	0.46	0.31-0.67	<0.0001
Piperonyl butoxide-PY LLIN arm	2352	18373	7.8	0.61	0.39-0.95	0.0273	26385	11.2	0.56	0.38-0.81	0.0020
Pyriproxyfen-PYLLIN arm	2352	24692	10.5	0.63	0.41-0.99	0.0431	38628	16.4	0.63	0.43-0.92	0.0150

The intervention arm is compared to the standard-PY LLIN arm at each time point. LLIN=long-lasting insecticidal net. PY=Pyrethroid. DR=Density reduction ratio. DRs are adjusted for baseline cluster-level variables used in restricted randomization. *EIR are weighted to account for the proportion of mosquitoes sampled to be tested for sporozoites. We have applied a Bonferroni correction for multiplicity given the multiple comparison arms and *a p value <0·017 was considered statistically significant.

S4: Trends in female Culex species density distribution over time across the study arm

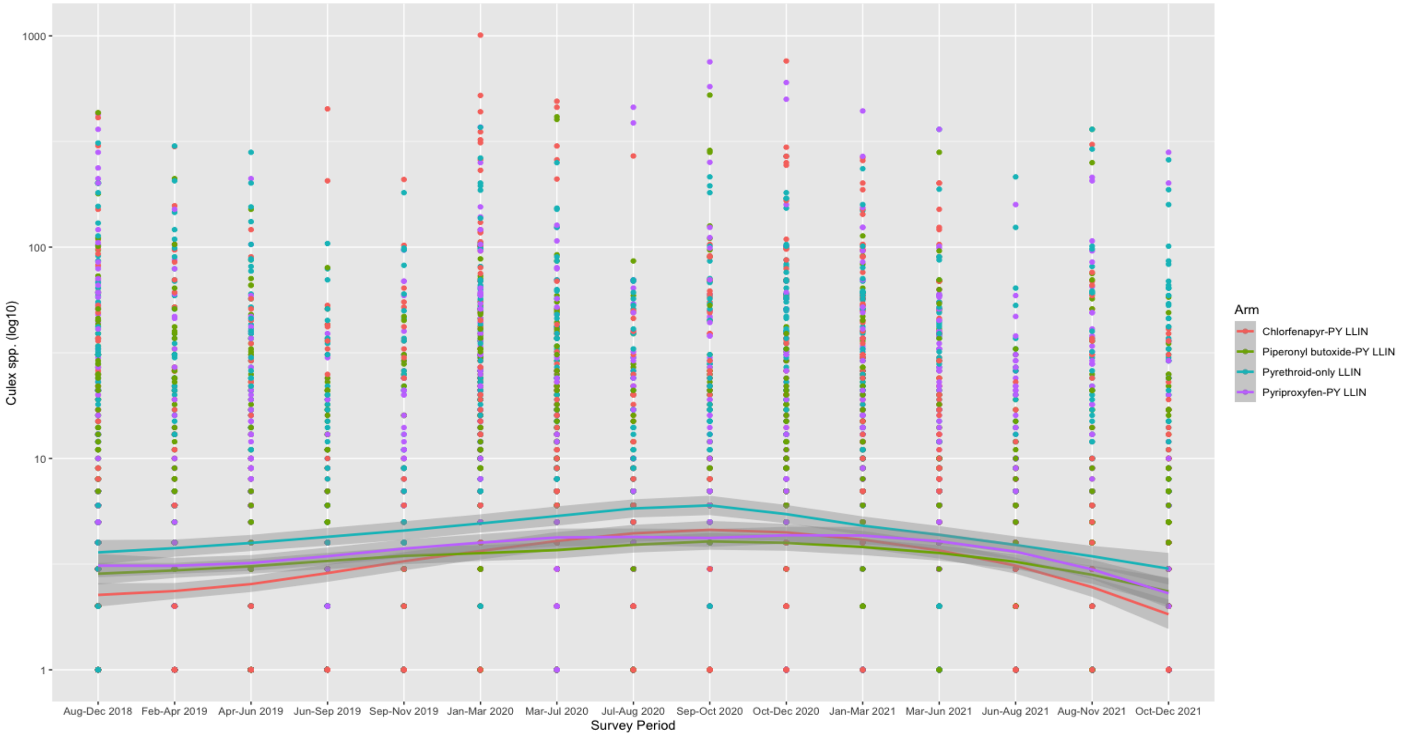

Plots showing smoothed averages female *Culex* population density over time across the treatment allocation arms defined as the orange (Chlorfenapyr-PY LLIN), green (Piperonyl butoxide-PY LLIN), blue (Pyriproxyfen-PY LLIN), and purple (Standard-Pyrethroid LLIN) with their respective 95% confidence intervals portrayed as grey shading. The plots were generated in R version 4.1.3 (R Development Core Team) using the “ggplot” packages

S5: Spatial differences of effectiveness of dual-AI LLINs

Table: Spatial heterogeneity of effectiveness of dual-AI LLINs on malaria vectors species

	**Number of households analysed**	**An. vector species**	**Density / night/ HH**	**DR**	**95%CI**	**p value****	***An. funestus s.l***	**Density / night/ HH**	**RR**	**95%CI**	**p value****	***An. gambiae s.l***	**Density / night/ HH**	**DR**	**95%CI**	**p value****
**Southern clusters**																
Pyrethroid-only LLIN arm (ref)	1118	7643	6.8	1			5801	5.2	1			1842	1.7	1		
Chlorfenapyr-PY LLIN arm	1008	3353	4.3	0.30	0.19-0.50	<0.0001	1685	2.2	0.21	0.12-0.38	<0.0001	1668	2.1	0.63	0.33-1.20	0.4301
Piperonyl butoxide-PY LLIN arm	784	2953	3.8	0.46	0.27-0.80	0.0057	1992	2.5	0.45	0.24-0.86	0.0152	961	1.2	0.72	0.35-1.49	0.3825
Pyriproxyfen-PY LLIN arm	783	7433	7.4	0.47	0.25-0.90	0.0229	5483	5.4	0.45	0.21-0.97	0.0420	1950	1.9	0.71	0.30-1.67	0.1565
**Northern clusters**																
Pyrethroid-only LLIN arm (ref)	1230	2650	2.2	1			1503	1.2	1			1147	0.9	1		
Chlorfenapyr-PY LLIN arm	1568	3828	2.4	0.60	0.39-0.93	0.0213	1213	0.8	0.37	0.22-0.63	<0.0001	2615	1.7	0.93	0.53-1.66	0.8158
Piperonyl butoxide-PY LLIN arm	1568	3225	2.1	0.67	0.44-1.02	0.0594	1329	0.9	0.54	0.33-0.88	0.0141	1896	1.2	0.88	0.50-1.53	0.6421
Pyriproxyfen-PY LLIN arm	1344	3520	2.6	1.22	0.78-1.89	0.3873	1657	1.2	1.15	0.68-1.94	0.6117	1863	1.4	1.33	0.74-2.40	0.3378

The intervention arm is compared to the standard-PY LLIN arm at each time point. LLIN=long-lasting insecticidal net. PY=Pyrethroid. DR=Density reduction ratio. DRs are adjusted for baseline cluster-level variables used in restricted randomization. *EIR are weighted to account for the proportion of mosquitoes sampled to be tested for sporozoites. We have applied a Bonferroni correction for multiplicity given the multiple comparison arms and *a p value <0·017 was considered statistically significant.

S6: Anopheles species composition

The figure shows Anopheles sibling species composition at baseline and each time point post-intervention

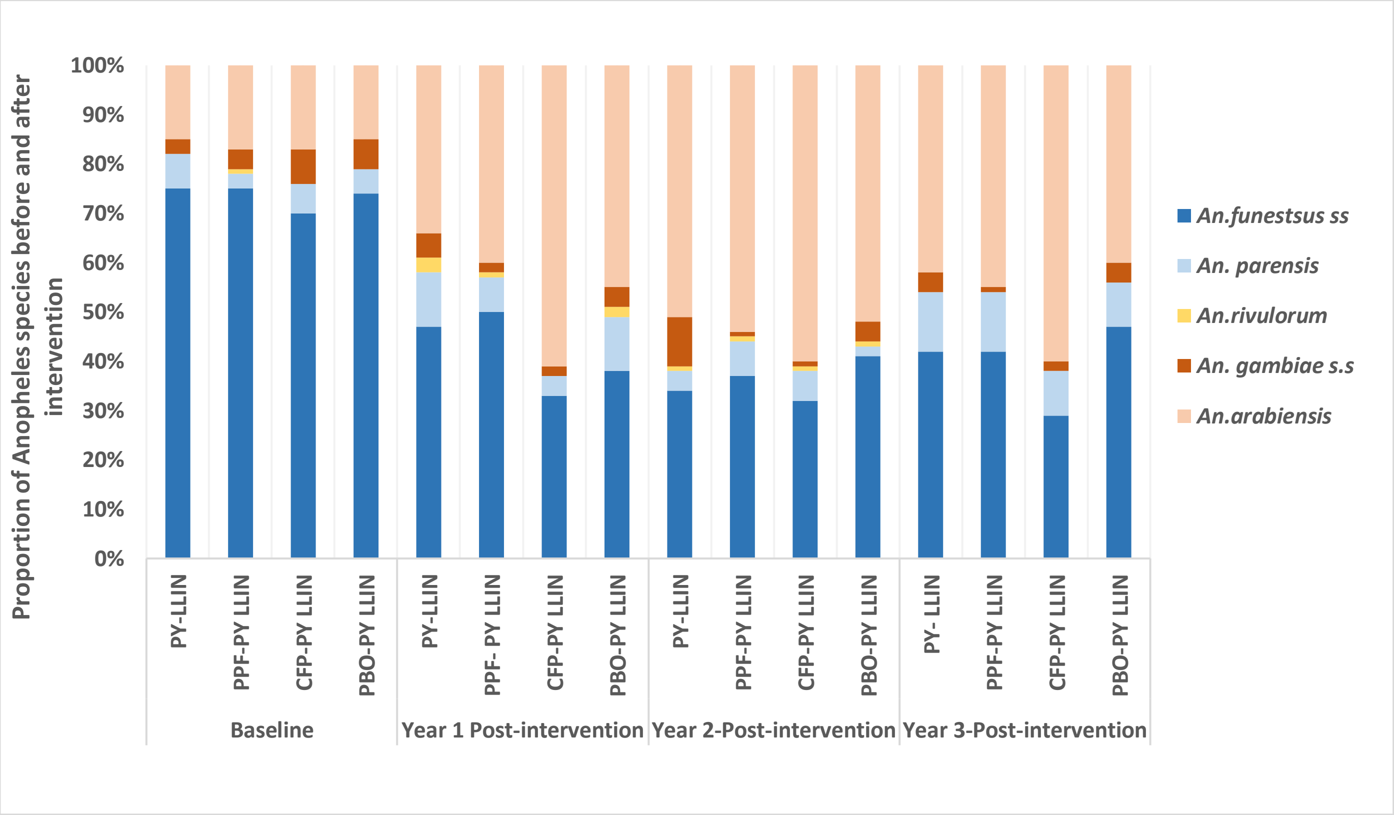

S7: Maxent model

Table: Model validation statistics for Maxent species distribution models of *An. funestus* and *An. arabiensis* comparing pilot predicted species distribution to post-intervention species occurrence by study arm and year.

Species	Validation test dataset	Arm	AUC	Standard error of AUC	Fractional predicted area	Test omission rate
An. funestus	Baseline	n/a	0.788	0.017	0.59	0.078
	Year 1	Pyrethroid-only LLIN arm (ref)	0.809	0.014	0.59	0.075
	Year 1	Chlorfenapyr-PY LLIN arm	0.792	0.019	0.59	0.038
	Year 1	Pyriproxyfen-PY LLIN arm	0.852	0.013	0.59	0.045
	Year 1	Piperonyl butoxide-PY LLIN arm	0.874	0.011	0.59	0.018
	Year 2	Pyrethroid-only LLIN arm (ref)	0.781	0.011	0.59	0.086
	Year 2	Chlorfenapyr-PY LLIN arm	0.759	0.012	0.59	0.061
	Year 2	Pyriproxyfen-PY LLIN arm	0.835	0.01	0.59	0.044
	Year 2	Piperonyl butoxide-PY LLIN arm	0.875	0.007	0.59	0.011
An. arabiensis	Baseline	n/a	0.8	0.023	0.684	0.025
	Year 1	Pyrethroid-only LLIN arm (ref)	0.812	0.023	0.684	0.058
	Year 1	Chlorfenapyr-PYLLIN arm	0.734	0.026	0.684	0.029
	Year 1	Pyriproxyfen-PY LLIN arm	0.729	0.023	0.684	0.014
	Year 1	Piperonyl butoxide-PY LLIN arm	0.747	0.024	0.684	0
	Year 2	Pyrethroid-only LLIN arm (ref)	0.73	0.019	0.684	0.051
	Year 2	Chlorfenapyr-PY LLIN arm	0.731	0.02	0.684	0.079
	Year 2	Pyriproxyfen-PY LLIN arm	0.756	0.017	0.684	0.021
	Year 2	Piperonyl butoxide-PY LLIN arm	0.708	0.02	0.684	0.081

The fractional predicted area and test omission rate are based on the minimum training presence threshold (0.102 for *An. funestus* and 0.155 for *An. arabiensis*). A p-value<0.05 indicates that test points are better predicted by the model than by random prediction with the same fractional predicted area.
